# Monosodium Urate Crystals Activate the Inflammasome in Primary Progressive Multiple Sclerosis

**DOI:** 10.3389/fimmu.2018.00983

**Published:** 2018-05-04

**Authors:** Federica Piancone, Marina Saresella, Ivana Marventano, Francesca La Rosa, Maria Antonia Santangelo, Domenico Caputo, Laura Mendozzi, Marco Rovaris, Mario Clerici

**Affiliations:** ^1^Laboratory of Molecular Medicine and Biotechnology, Don Gnocchi Foundation, IRCCS, Milan, Italy; ^2^Laboratory of Clinical Analysis, Don Gnocchi Foundation, IRCCS, Milan, Italy; ^3^Department of Neurology, Don Gnocchi Foundation, IRCCS, Milan, Italy; ^4^Department of Physiopathology and Transplants, University of Milano, Milan, Italy

**Keywords:** multiple sclerosis, inflammasome, uric acid, neuroinflammation, immunity

## Abstract

Inflammasome-driven inflammation is postulated to play a role in multiple sclerosis (MS), but there is no direct evidence that the nod-like receptor protein 3 (NLRP3) inflammasome is involved in MS pathogenesis. Uric acid was shown to be one of the “danger” signals involved in the activation of NLRP3 inflammasome; notably, the concentration of uric acid is increased in the serum and in the cerebrospinal fluid of MS individuals. To better investigate the role of the NLRP3 inflammasome in MS-associated inflammation, we primed with lipopolysaccharide and stimulated with monosodium urate crystals PBMCs of 41 MS patients with different disease phenotypes. Eleven individuals with primary progressive MS (PPMS), 10 individuals with stable relapsing–remitting MS (SMS), 10 individuals with acute relapsing–remitting MS (AMS), 10 individuals with benign MS were analyzed; 10 healthy controls were enrolled as well in the study. The expression of the NLRP3, apoptosis-associated speck-like protein containing CARD (ASC), caspase-1, caspase-8, IL-1β, and IL-18 inflammasome genes was evaluated by RT-PCR. NLRP3 and ASC-speck protein expression was analyzed by FlowSight AMNIS, whereas production of the pro-inflammatory cytokines IL-1β and IL-18 and of caspase-1 and caspase-8 was measured by ELISA in supernatants. Results showed that uric acid serum concentration was significantly increased in PPMS; in these and in AMS patients, mRNA for NLRP3, ASC, and IL-18 was upregulated as well, but caspase-8 mRNA was upregulated only in PPMS. Expression of NLRP3 and ASC-speck protein was significantly increased in PPMS, SMS, and AMS patients, but IL-18 and caspase-8 production was significantly increased only in PPMS, in whom a direct correlation between hyperuricemia and caspase-8 was detected. The NLRP3/caspase-8 inflammasome pathway is activated in PPMS, possibly as a consequence of hyperuricemia. Therapeutic strategies reducing NLRP3 activation and/or lowering hyperuricemia could be useful in the therapy of PPMS.

## Introduction

Multiple sclerosis (MS), the most common non-traumatic disabling neurological disease in young adults, is an inflammatory and demyelinating disease of the central nervous system (CNS) that is mediated by multiple immune effector mechanisms. From a pathogenic point of view, MS is a heterogeneous condition consisting of neuroinflammatory, autoimmune, and neurodegenerative processes that are the consequence of an inappropriate activation of the immune system toward myelin “self” antigens.

Similarly to other neurodegenerative diseases, MS is also characterized by complex biochemical alterations affecting neuronal functions. Uric acid, the product of purine catabolism, is a damage-associated molecular pattern (DAMP) released from dying cells whose concentration is reported to be elevated in the cerebrospinal fluid (CSF) and the serum of MS patients, in particular, in the chronic phase of the disease (1, 2). Notably, though, even if a correlation has been demonstrated between serum concentration of uric acid and susceptibility to the disease, hyperuricemia was not observed in every analyzed cohort of MS patients (3–8).

Uric acid activates the nod-like receptor protein 3 (NLRP3) inflammasome (9), an event suggested to be involved in the pathogenesis of MS. Inflammasomes are signaling complexes that sense inflammatory signals and promote inflammation by maturation and release of the pro-inflammatory cytokines interleukin (IL)-1β and IL-18. Classically, functional inflammasome complexes are composed of three proteins: a sensor (NLRP3, NLRP1, NLRC4, AIM2), an adaptor [apoptosis-associated speck-like protein containing CARD (ASC)], and catalytic proteins (pro-caspase-1, pro-caspase-8). Upon detecting inflammatory signals, the inflammasome sensor molecule induces a rapid polymerization of the adaptor protein ASC into large helical filaments (“specks”), which represent the hallmark of inflammasome activation (10). The assembly of the three proteins results in the generation of a complex that mediates the self-cleavage of pro-caspase-1 and pro-caspase-8 to their active analogs. Caspase-1 and caspase-8 will then cleave the immature forms of the pro-inflammatory cytokines IL-1β and IL-18 into the mature bioactive forms.

Among the inflammasomes, much attention has been given to the NLRP3 complex due to its potential contribution to several diseases, including neurodegenerative conditions. The NLRP3 inflammasome can be activated by a wide range of stimulators, including pathogens, viruses, bacteria, extracellular ATP, amyloid β and uric acid (9, 11, 12). In experimental autoimmune encephalomyelitis (EAE), in particular, the most widely investigated animal model of MS, activation of the NLRP3 inflammasome was shown to have a critical role. Thus, augmented levels of caspase-1, IL-1β, and IL-18 are observed in the pathogenesis of EAE (13) and, conversely, the absence of the *NLRP3* gene results in diminished Th1 and Th17 encephalitogenic responses and reduces the inflammatory infiltrate in the spinal cord (14). Very recent results, in particular, show that, in caspase-1-deficient mice models, the processing and the release of IL-1β can be taken up by caspase-8 (15). These data indicate that caspase-8, together with ASC and NLRP3, can drive IL-1β production in EAE and show the presence of a caspase-1-independent form of EAE (16). These results also suggest that caspase-1 activation may be not be present in all phenotypes of MS, which is a clinically heterogeneous disease.

In MS, it was shown that the upregulation of caspase-1, IL-1β, and IL-18 associates with the progression and severity of disease (17–19). Caspase-1 expression, in particular, is elevated in MS plaques (20) and, together with that of IL-18, in peripheral mononuclear cells of MS patients (21). Notably, whereas augmented serum and CSF IL-18 concentration was shown to be present in MS individuals (17, 22), not all authors have found increases of IL-1β in the CSF of these patients (23–26). IL-1β is known to promote the differentiation of naive CD4+ T cells into Th17 T lymphocytes (27, 28), whereas IL-18 is a potent activator of polarized Th1 cells for IFNγ production and lymphocytes proliferation (28). These observations are important within the pathogenesis of MS as both Th1 and Th17 T lymphocytes have been implicated in the pathology of the disease (29–32).

To verify whether NLRP3 activation could be detected in MS patients with different disease phenotypes, and to determine if hyperuricemia drives NLRP3 activation in MS, we analyzed uric acid serum concentration and NLRP3 inflammasome activation in MS patients affected by active MS or with a clinically quiescent disease.

## Materials and Methods

### Patients and Controls

This study was approved by and carried out in accordance with the guidelines of the ethic committee of the Don Gnocchi Foundation and conformed to the Declaration of Helsinki. All participants gave informed consent according to a protocol approved by the local ethics of the Don Gnocchi Foundation.

Forty-one patients affected by MS as diagnosed by clinical and laboratory parameters, and followed by the Centro Sclerosi Multipla of the Don Gnocchi Foundation in Milano, Italy, were included in the study. Twenty patients (13 females and 7 males) were diagnosed as been affected by relapsing–remitting (RR) MS with or without sequelae. The disease had been clinically stable in 10 patients for at least 6 months prior to the study period; these patients (mean age = 45 ± 11 years; range = 26–62 years; 7 females and 3 males) were classified as patients with stable relapsing-remitting MS (SMS). The diagnosis of SMS was confirmed by brain and spinal cord magnetic resonance imaging (MRI) with gadolinium: MRI showed no areas of enhancement at the time of enrollment. Mean disease duration was 20 ± 10 years (range = 1–31 years); the median Kurtze Expanded Disability Status Scale (EDSS) score was 4.5 (range = 4–9). Ten other RRMS patients (mean age = 46 ± 13 years; range = 28–69 years; 6 females and 4 males) were undergoing clinical relapses of the disease and were classified as patients with acute relapsing-remitting MS (AMS). MRI scans performed during the acute phases showed enhancing lesions in all AMS patients. Mean disease duration was 19 ± 15 years (range = 4–39 years); the median EDSS was 4.25 (range = 2–6). Eleven patients (mean age = 60 ± 10 years; range = 48–72 years; 5 females and 6 males) were diagnosed as been affected by PPMS; the MRI evidenced a stability of the lesion load at the time of enrollment. Mean disease duration was 16 ± 10 years (range = 5–36 years); the median EDSS was 6 (range = 5.5–8). Finally, 10 patients (mean age = 62 ± 14 years; range = 50–68 years; 5 females and 5 males) were affected by benign MS (BMS); MRI showed stability or in many cases, an improvement of the lesion load at the time of enrollment. BMS was diagnosed based on the most widely accepted definition, i.e., an EDSS score ≤ 3.0 > 15 years from the clinical onset of disease. Mean disease duration was 31 ± 8 years (range = 24–39 years); the median EDSS score was 2 (range = 1–3). None of the patients had received immunosuppressive drugs in the year prior to the study period.

Ten sex and age matched healthy controls (HC) (mean age = 53 ± 5 years; range = 42–58 years; 8 females and 2 males) was enrolled as well in the study.

### Whole Blood and Serum Sample Collection and Cell Separation

Thirty milliliters of whole blood was collected in EDTA-containing vacutainer tubes (Becton Dickinson and Co., Rutherford, NJ, USA). PBMCs were separated on lymphocyte separation medium (Organon Teknika Corp., Durham, NC, USA) and washed twice in PBS. Leukocytes viability was determined using a Bio-Rad TC20 Automated Cell Counter (Bio-Rad, CA, USA). Serum was collected in vacutainer tubes containing serum separator (Becton Dickinson and Co.). After 40 min at room temperature, samples were centrifuged at 3,000 rpm for 10 min to separate sera.

### Uric Acid Concentration in Sera

Serum uric acid concentration was measured by the uricase-peroxidase method (Beckman Coulter Synchron LX, Beckman Coulter, Fullerton, CA, USA) following the manufacturer’s instructions.

### Cell Cultures: Human Monocytic THP-1 Cell Line and PBMCs

The human monocytic cell line THP-1 was provided by Istituto Zooprofilattico Sperimentale della Lombardia e dell’Emilia Romagna (Brescia, Italy) and maintained in RPMI 1640 supplemented with 10% fetal bovine serum, 2 mM l-glutamine, and 1% penicillin (Invitrogen Ltd., Paisley, UK) (medium) at 37°C in a humidified 5% CO_2_ atmosphere.

PBMCs were maintained in RPMI 1640 supplemented with 10% human serum, 2 mM l-glutamine, and 1% penicillin (Invitrogen Ltd., Paisley, UK) (medium) at 37°C in a humidified 5% CO2 atmosphere. THP-1 cells and PBMCs were resuspended at 1 × 10^6^/ml and were either: (1) cultured in medium alone (unstimulated); or (2) primed 2 h with lipopolysaccharide (LPS) (1 μg/ml) (Sigma-Aldrich, St. Louis, MO, USA); or (3) stimulated with 50, 100, or 200 µg/ml of monosodium urate crystals (MSU) for 22 h; and (4) primed 2 h with LPS and stimulated with 50, 100, or 200 µg/ml of MSU for 22 h at 37°C in a humidified 5% CO_2_ atmosphere (33). LPS pre-incubation is required because neither NLRP3 nor pro-IL-1β are constitutively expressed and require transcriptional induction (9, 11, 12). Each experiment was run at least in triplicate.

### Determination of the Optimal Dose of MSU to Be Used in Stimulation

THP-1 cells and PBMCs stimulated with different concentration of MSU were evaluated for vitality with (3-4,5-dimethylthiazol-2-yl-2,5-diphenyl-tretrazolium bromide) the MTT cell viability assay. Briefly, MTT dissolved in PBS was added to the cells (20 µl/well). Cells were incubated at 37°C for 22 h, centrifuged, pellets were dissolved using 100 μl/well of dimethyl sulfoxide, and plates were read in a micro plate reader using a test wavelength of 550 nm and a reference wavelength of 650 nm. Results were calculated as: % cytotoxicity = 100 − [optical density (OD) test − OD control]/OD control × 100. The concentration of 200 µg/ml of MSU was toxic to cells (>50% cell death); cell mortality was <5% using 100 µg/ml of MSU, a dose that optimally stimulated the NLRP3 inflammasome (see Results).

### RNA Extraction and Reverse Transcription

RNA was extracted from THP-1 cells or PBMCs using the acid guanidinium thiocyanate-phenol-chloroform method. RNA was dissolved in RNase-free water and purified from genomic DNA with RNase-free DNase (RQ1 DNase; Promega, Madison, WI, USA). One microgram of RNA was reverse transcribed into first-strand cDNA using RT2 First Strand kit (Qiagen, Hilden, Germany) according to manufacturer’s instruction.

### Real-Time RT-PCR

Quantitative real-time RT-PCR (qPCR) was performed on the Biorad CFX Real-Time PCR instrument (Biorad) using RT2 SYBR Green qPCR mastermix (Qiagen). All primers (NLRP3, ASC, caspase-1, caspase-8, IL-1β, IL-18) (Qiagen) were cDNA specific. All the samples were evaluated for glyceraldehyde 3-phosphate dehydrogenase (GAPDH) expression by real-time PCR to test the quality of RNA. Results were expressed as ΔΔCt (where Ct is the cycle threshold) and are presented as ratios between the target gene and the GAPDH housekeeping mRNA.

### Image Stream Analysis by FlowSight AMNIS

PBMCs (1 × 10^6^), stimulated as described above, were fixed with 100 µl of PFA (1%) (BDH, UK), permeabilized with 100 µl of Saponine (0.1%) (Life Science VWR, Lutterworth, Leicestershire, LE), and stained with FITC-anti human NLRP3 (Clone #768319, isotype Rat IgG2a, R&D Systems, Minneapolis, MN, USA) and PE-anti human ASC (clone HASC-71, isotype mouse IgG1, Biolegend, San Diego, CA, USA) for 1 h at room temperature; cells were then washed with PBS, centrifuged at 1,500 rpm for 10 min, resuspended in 50 µl of PBS, and examined using the AMNIS Flowsight Imaging. Results were analyzed by IDEAS analysis software (Amnis Corporation, Seattle, WA, USA).

The analysis of NLRP3 expression was performed by internalization feature utilizing a mask representing the whole cell, defined by the brightfield image, and an internal mask defined by eroding the whole cell mask.

Apoptosis-associated speck-like protein containing CARD speck formation was analyzed using the same mask of internalization feature, differentiating diffuse or spot (speck) fluorescence inside of cells. Threshold mask was used to separate all ASC positive cells population in ASC-Speck spot cells or ASC-diffuse cells by the different diameter of the spot area: in ASC-speck, the spot shows a small area and high max pixel *vice versa* in cell with ASC-diffuse.

### ELISA

IL-1β, IL-18, caspase-1, and caspase-8 concentration was determined by ELISA according to the manufacturer’s recommendations (Quantikine Immunoassay; R&D Systems) in supernatants from unstimulated or stimulated THP-1 cells and PBMCs. The wells were read on a plate reader (Sunrise, Tecan, Mannedorf, Switzerland) and optical density (OD) was determined at 450/620 nm. The measured absorbance is proportional to the concentration of cytokines (IL-1β and IL-18) or caspases (caspase-1 and caspase-8) present in the supernatants expressed in picogram per milliliter and calculated by dividing OD measurement generated from the assay by OD cut-off calibrator. All the experiments were performed in triplicate.

### Statistical Analysis

Quantitative data were not normally distributed (Shapiro–Wilk test) and are thus summarized as median and interquartile range. Comparisons between groups were analyzed used a Kruskal–Wallis ANOVA for each variable. Comparisons among the different groups were made using a 2-tailed Mann–Whitney *U* test performed for independent samples. Acid uric serum data were normally distributed and were summarized as mean ± SD. In this case, comparisons were performed using ANOVA and unpaired Student’s *t*-test. The statistical correlations between uric acid serum concentration and caspase-8 protein were investigated using Spearman correlation coefficient and 95% confidence limits performed by Fisher’s *Z* transformation.

Data analysis was performed using the MedCalc statistical package (MedCalc Software bvba, Mariakerke Belgium). *p-*Values of less than 0.05 were considered statistically significant.

## Results

### Uric Acid Serum Concentration

**Figure 1:**
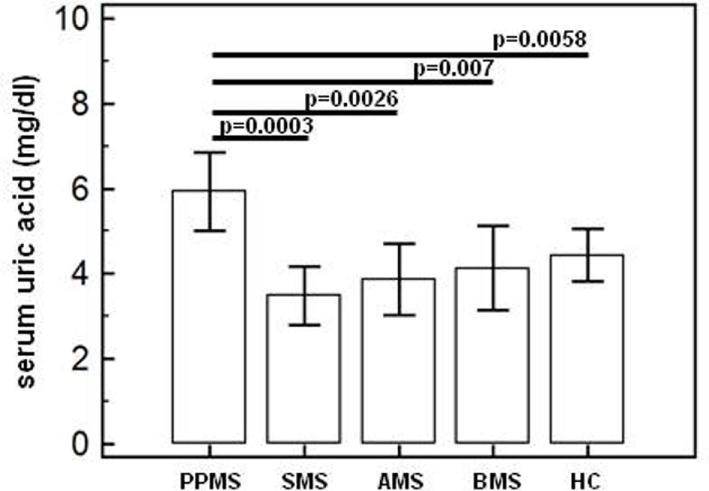
Serum uric acid concentration. Uric acid concentration (means; mg/dl) in serum of primary progressive (PP, *n* = 11), acute relapsing-remitting (A, *n* = 10), stable relapsing-remitting (S, *n* = 10), or benign (B, *n* = 10) multiple sclerosis (MS) patients and healthy controls (HC, *n* = 10). SDs are indicated by vertical bars. Statistical significance is shown.

Serum acid uric concentration was significantly increased in PPMS (mean = 6.0 mg/dl ± 1.8) compared to the values observed in all others MS patients (AMS = 3.86 mg/dl ± 0.79, SMS = 3.48 mg/dl ± 0.74, BMS = 4.13 mg/dl ± 1.17) and in HC (4.23 mg/dl ± 0.61) (*p* ≤ 0.005 for all comparison) (Figure 1).

### Modulation of Inflammasome Genes in LPS-Primed and MSU-Stimulated THP-1 Cells and in PBMCs

**Figure 2:**
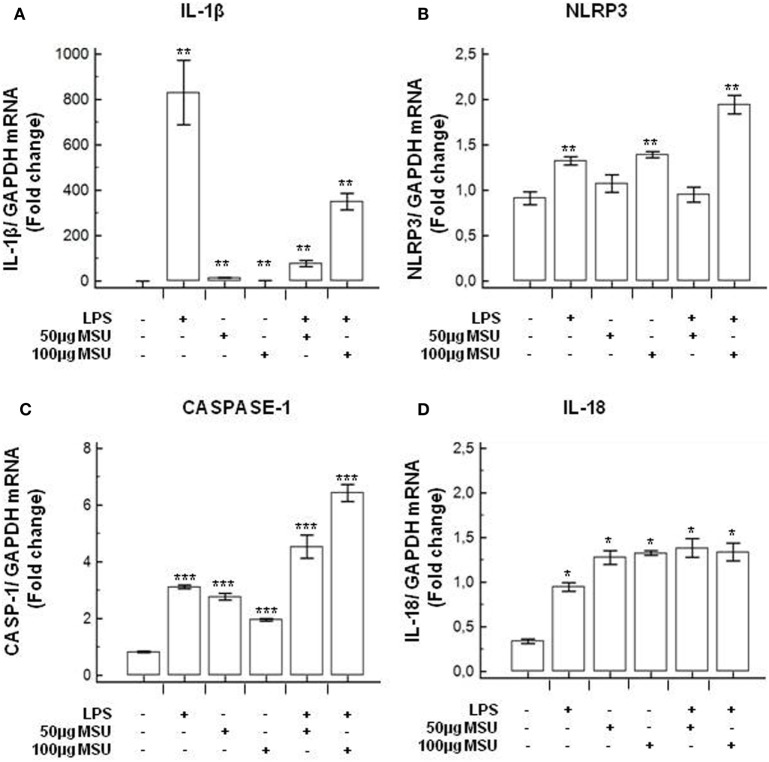
Modulation of inflammasome genes by monosodium urate crystals (MSU) in THP1 cells. THP-1 cells were treated with lipopolysaccharide (LPS) (1 µg/ml) for 2 h and/or with the indicate concentration of MSU crystals for 22 h. RNA was isolated from THP-1 cells and the level of IL-1β **(A)**, Nod-like receptor protein 3 (NLRP3) **(B)**, Caspase-1 **(C)**, and IL-18 **(D)** transcription was determined using SYBR green qPCR. Human GAPDH was used for normalization.

**Figure 3:**
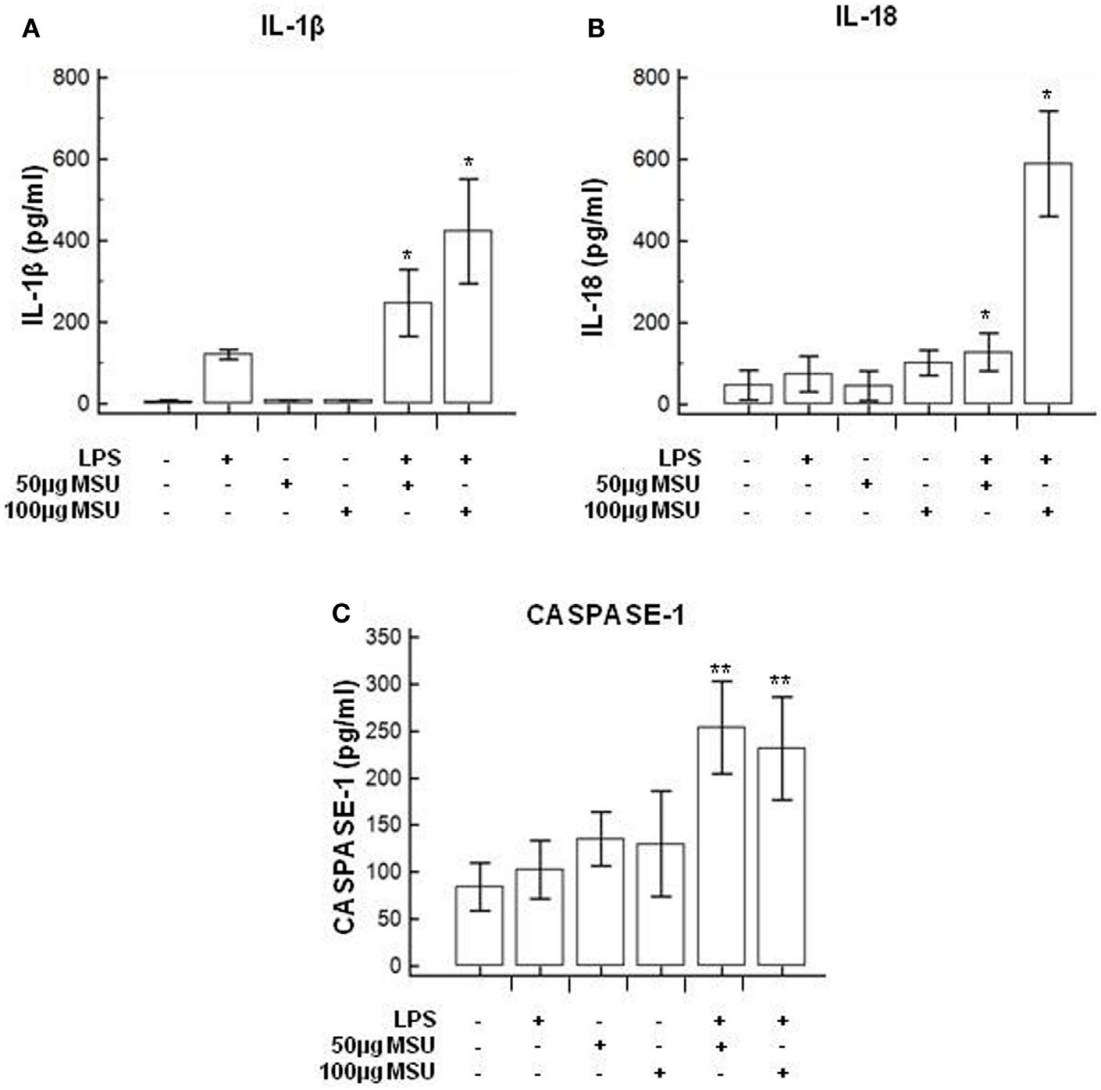
Modulation of inflammasome effector proteins by monosodium urate crystals (MSU) in THP1 cells. THP-1 cells were treated with lipopolysaccharide (LPS) (1 µg/ml) for 2 h and/or with the indicate concentration of MSU crystals for 22 h. The supernatants of cell cultures were collected and IL-1β **(A)**, IL-18 **(B)**, and caspase-1 **(C)** release was measured by ELISA. Data are representative of three independent experiments and expressed as means ± SD. *p < 0.05, **p < 0.01, ***p < 0.001 significant difference from cells untreated.

**Figure 4:**
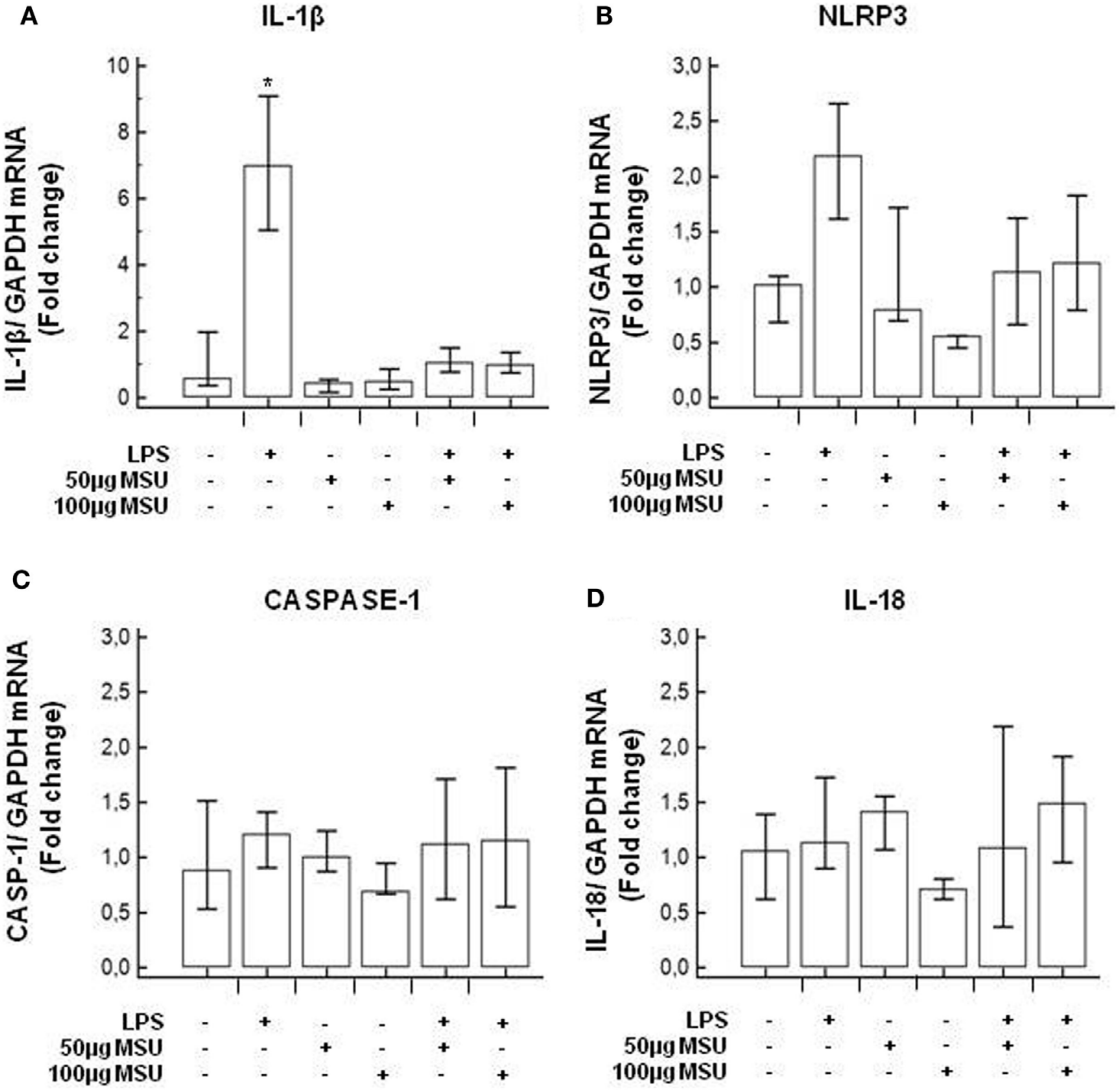
Modulation of inflammasome genes by monosodium urate crystals (MSU) in PBMCs of healthy control (HC) subjects. PBMCs of HC were stimulated with lipopolysaccharide (LPS) (1 µg/ml) for 2 h and/or with the indicate concentration of MSU crystals for 22 h. RNA was isolated from PBMCs and IL-1β **(A)**, Nod-like receptor protein 3 (NLRP3) **(B)**, caspase-1 **(C)**, and IL-18 **(D)** were determined using SYBR green qPCR. Human GAPDH was used for normalization.

**Figure 5:**
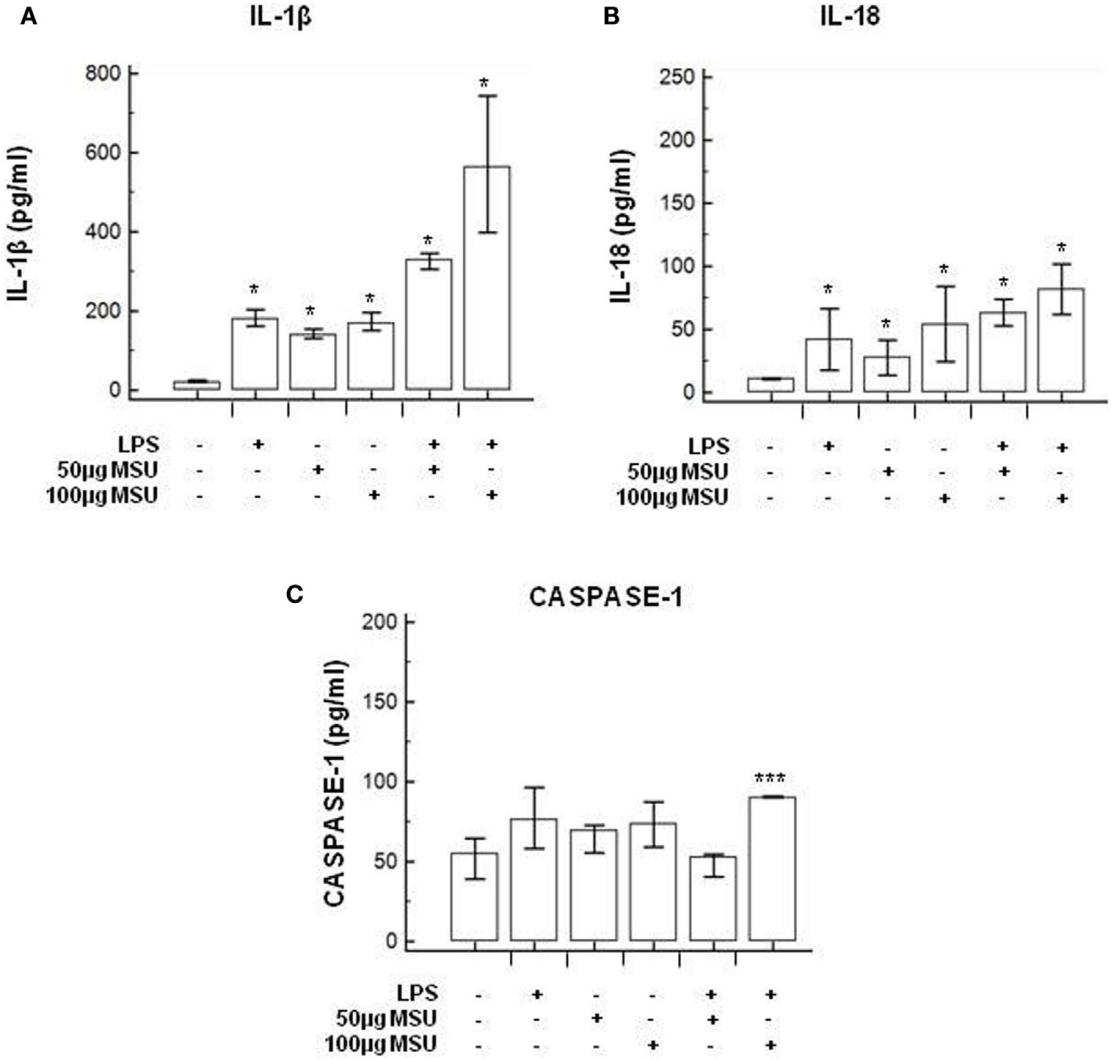
Modulation of inflammasome effector proteins by monosodium urate crystals (MSU) in PBMCs of HC subjects. PBMCs of HC subjects were stimulated with lipopolysaccharide (LPS) (1 µg/ml) for 2 h and/or with the indicate concentration of MSU crystals for 22 h. IL-1β **(A)**, IL-18 **(B)**, and caspase-1 **(C)** were quantified in supernatants by ELISA. Data are representative of three independent experiments and expressed as means ± SD. *p < 0.05, **p < 0.01, ***p < 0.001 significant difference from cells untreated.

To verify whether MSU stimulates the assembly of functional inflammasome complexes and to determine the optimal dose of MSU to be used in cell cultures, mRNA expression of NLRP3, caspase-1, IL-1β, and IL-18 was evaluated in THP-1 cells and in PBMCs of HC. Cells were unstimulated; stimulated with LPS alone; stimulated with 50, 100, or 200 µg/ml of MSU alone; or LPS-primed and stimulated with 50, 100, or 200 µg/ml of MSU. Results showed that the highest dose of MSU was toxic to the cells (mortality > 50%). The two lower doses of MSU positively modulated these genes but a consistent upregulation of inflammasome related genes was observed in THP-1 cells stimulated with 100 µg/ml of MSU alone (Figure 2). This was further confirmed by measuring IL-18, caspase-1, and IL-1β concentration in supernatants of THP-1 cells (Figure 3). Results obtained when PBMCs of HC individuals were analyzed showed that the expression of inflammasome related genes (Figure 4), as well as the production of the inflammasome effector proteins (Figure 5) were only marginally upregulated. Based on these results, and on the lack of toxicity (cell death <5%), the concentration of 100 µg/ml MSU was used to stimulate PBMCs obtained from patients.

### Modulation of Inflammasome Genes in LPS-Primed and MSU-Stimulated PBMCs of MS Subjects

mRNA expression of NLRP3, ASC, caspase-1, caspase-8, IL-1β, and IL-18, genes involved in the assembly, the activation, and the downstream signaling of inflammasomes was quantified by qPCR in all MS patients and controls. Data are expressed as the fold change (nFold) comparing results observed in unstimulated cells (medium) to those obtained in cells primed with LPS and stimulated with MSU 100 µg/ml.

Results showed that, whereas inflammasome protein genes were not modulated in LPS-primed and MSU-stimulated PBMCs of HC and BMS individuals, these genes were significantly upregulated in cells of PPMS, AMS, and SMS patients. Notably, though the functional triad of inflammasome receptor (NLRP3), effector (ASC), and catalytic (caspase-8) genes was upregulated by MSU in PPMS alone: thus, the assembly of a potentially functional inflammasome complex was stimulated by MSU only in cells of MS patients with a primary progressive form of disease.

**Figure 6:**
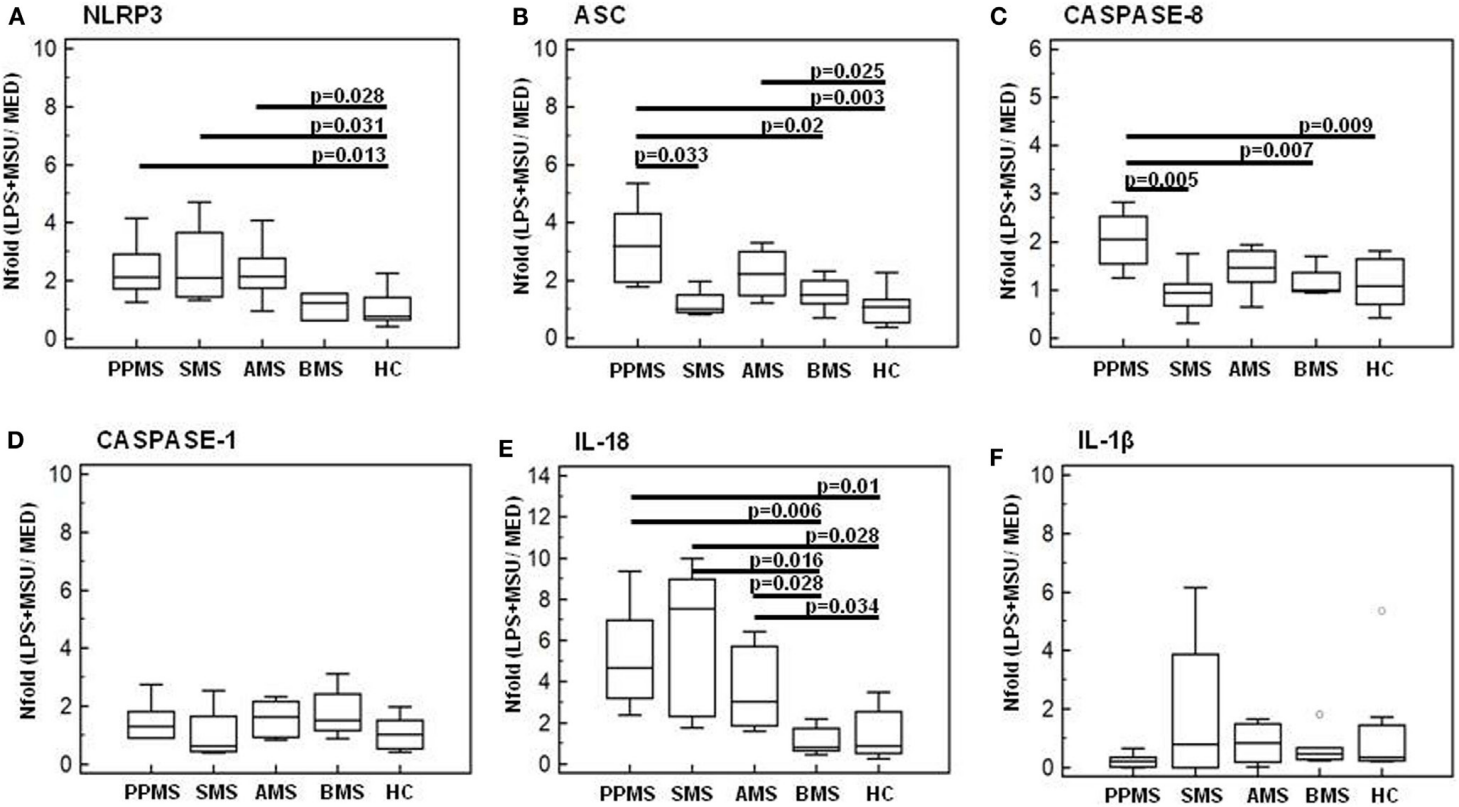
Genes of inflammasome proteins in lipopolysaccharide (LPS)-primed and monosodium urate crystals (MSU)-stimulated PBMCs. mRNA expression by real-time PCR. Single real-time PCR results obtained in LPS-primed and MSU-stimulated immune cells of primary progressive (PP, *n* = 11), acute relapsing-remitting (A, *n* = 10), stable relapsing-remitting (S, *n* = 10), or benign (B, *n* = 10) Multiple sclerosis (MS) patients and healthy controls (HC, *n* = 10). Nod-like receptor protein 3 (NLRP3) **(A)**, ASC **(B)**, caspase-8 **(C)**, caspase-1 **(D)**, IL-18 **(E)**, and IL-1β **(F)** mRNA levels are shown. The results are indicated as fold-change expression from the unstimulated samples. Summary results are shown in the bar graphs. The boxes stretch from the 25 to the 75 percentile; the line across the boxes indicates the median values; the lines stretching from the boxes indicate extreme values. Statistical significance is shown.

To summarize: (1) NLRP3 gene expression was significantly upregulated (nFold > 2) in PPMS, AMS, and SMS compared to HC (*p* < 0.05) (Figure 6A); (2) ASC was significantly upregulated in PPMS (nFold > 3.2) and AMS (nFold > 2.2) compared to all other groups of MS patients and HC (*p* < 0.005) (Figure 6B); and (3) caspase-8 expression was upregulated in PPMS (nFold > 2.5) compared to all other groups of MS patients and HC (*p* < 0.05 in all cases) (Figure 6C). Notably, no statistically significant differences were detected when caspase-1 mRNA expression was analyzed (Figure 6D). These results seem to confirm that the inflammasome activity follows a caspase-1-independent pathway in PPMS-associated inflammation.

IL-1β and IL-18 gene expression was analyzed next in LPS-primed and MSU-stimulated PBMCs of all individuals. Results showed that IL-18 gene expression was significantly increased in PPMS (nFold > 4.6), AMS (nFold > 3), and SMS (nFold > 7.5) compared to BMS and HC (*p* < 0.05 for all comparison) (Figure 6E). No statistically significant differences were detected when IL-1β mRNA expression was analyzed (Figure 6F).

### NLRP3 Production and ASC-Speck Formation in LPS-Primed and MSU-Stimulated PBMCs

**Figure 7:**
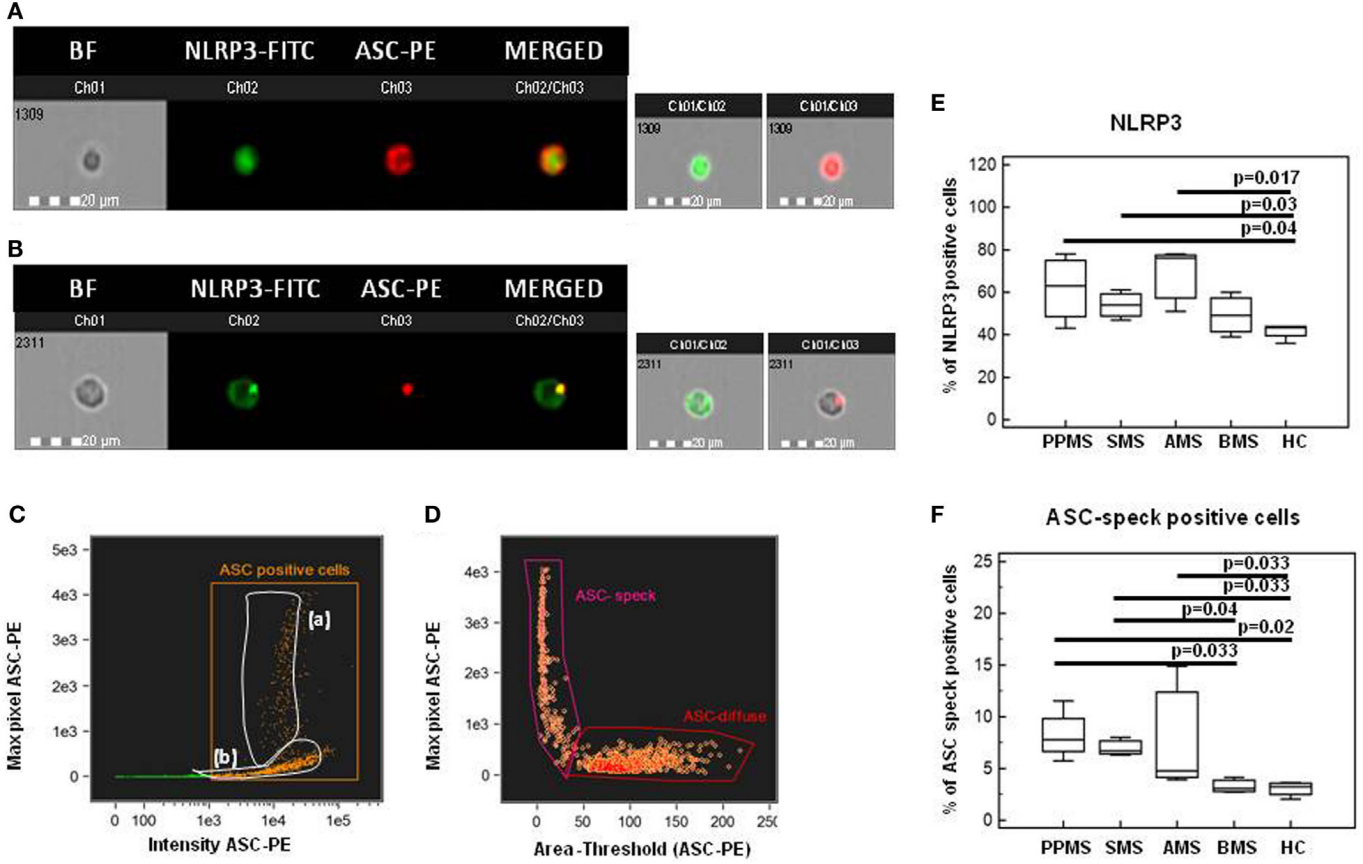
Nod-like receptor protein 3 (NLRP3) expression and apoptosis-associated speck-like protein containing CARD (ASC)-speck formation in lipopolysaccharide (LPS)-primed and monosodium urate crystals (MSU)-stimulated PBMCs. Representative images of NLRP3 expression and ASC-speck formation in LPS-primed and MSU-stimulated PBMCs. [**(A)**, ASC-diffuse; **(B)**, ASC-speck]. The first column shows cells in brightfield (BF), second column shows NLRP3-FITC fluorescence, third column shows ASC-PE fluorescence, and fourth column shows florescence of ASC merged with NLRP3 (IDEA software). The percentage of ASC speck positive cells was performed using the same mask of internalization feature **(C)**, differentiating spot (speck) or diffuse fluorescence inside of cells (DF): threshold mask was used to separate all ASC positive cells population in ASC-speck spot cells or ASC-diffuse cells by the different diameter of the spot area **(D)**. In ASC-speck cell, the spot shows a small area and high max pixel, conversely, in ASC-diffuse cell, the fluorescence shows a large area and low max pixel. Summary results of NLRP3 **(E)** and ASC-speck positive cells **(F)** in LPS-primed and MSU-stimulated PBMCs of PPMS, SMS, AMS, BMS, and HC are shown in the bar graphs. The boxes stretch from the 25 to the 75 percentile; the line across the boxes indicates the median values; the lines stretching from the boxes indicate extreme values.

Nod-like receptor protein 3 production and ASC-speck formation were investigated next by Flowsight AMNIS analyses in LPS-primed and MSU-stimulated PBMCs of all patients and controls. Representative images are provided in Figures 7A–D.

Results from NLRP3 protein expression analysis confirmed that the percentage of cells expressing NLRP3 was significantly increased in PBMCs of PPMS, SMS, and AMS compared to those of HC (*p* < 0.05 for all comparison) (Figure 7E).

Apoptosis-associated speck-like protein containing CARD-speck formation, the hallmark of inflammasome activation, was then analyzed by Flowsight AMNIS in LPS-primed and MSU-stimulated PBMCs of all patients and controls. Results showed that the percentage of ASC-speck positive PBMCs was significantly increased in PPMS, SMS and AMS compared to BMS and HC (*p* < 0.05 for all comparison) (Figure 7F). Taken together, these results confirmed those obtained by gene expression analysis.

### Caspase-1, Caspase-8, and Inflammasome Effector Cytokines Production by LPS-Primed and MSU-Stimulated-PBMCs

**Figure 8:**
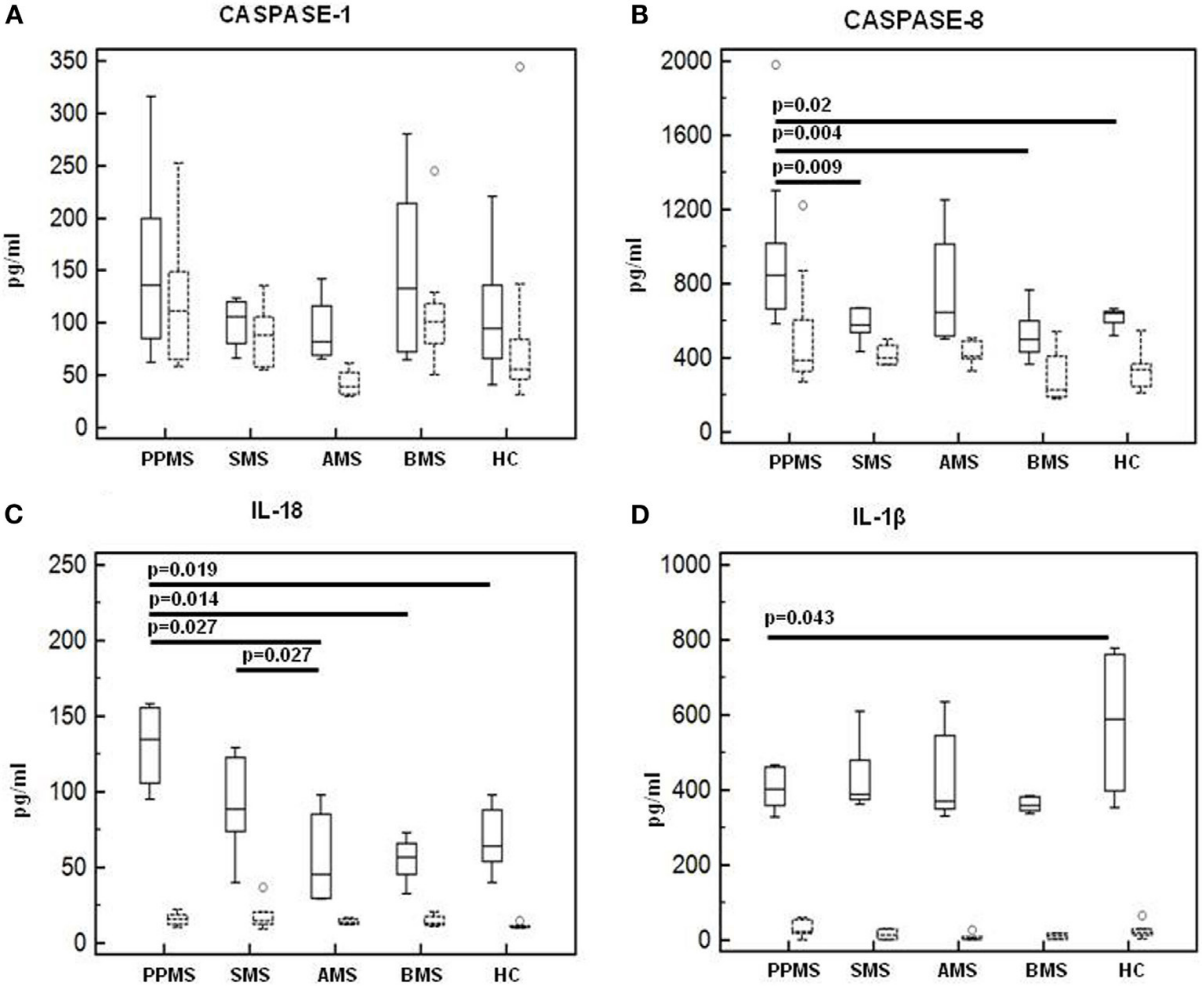
Caspase-1, Caspase-8, and inflammasome effector cytokines production in lipopolysaccharide (LPS)-primed and monosodium urate crystals (MSU)-stimulated-PBMCs. Caspase-1 **(A)**, Caspase-8 **(B)**, interleukin-18 **(C)**, and IL-1β **(D)** production was assessed by multiplex ELISA in supernatants of unstimulated and LPS-primed and MSU-stimulated cells of primary progressive (PP, *n* = 11), acute relapsing-remitting (A, *n* = 10), stable relapsing-remitting (S, *n* = 10), or benign (B, *n* = 10) multiple sclerosis (MS) patients and healthy controls (HC, *n* = 10). Summary results are shown in the bar graphs. Solid lines represent supernatants from LPS-primed and MSU-stimulated cells. Dashed lines indicate supernatants from unstimulated cells. The boxes stretch from the 25 to the 75 percentile; the line across the boxes indicates the median values; the lines stretching from the boxes indicate extreme values.

Caspase-1 and caspase-8 production by LPS-primed and MSU-stimulated cells was evaluated next in all patients and controls. Results showed that, whereas caspase-1 production was similar in all the groups of individuals (Figure 8A), caspase-8 production was significantly increased in PPMS (median = 846 pg/ml) alone compared to all other groups (Figure 8B). IL-1β and IL-18 production was analyzed as well in LPS-primed and MSU-stimulated PBMCs. Once again, IL-18 was significantly increased in PPMS (median = 134 pg/ml) alone (Figure 8C). IL-18 production was not increased in AMS or SMS patients despite the upregulation of IL-18 mRNA that was detected in these individuals. This apparently puzzling result could be explained by the observation that, even if IL-18 mRNA levels are increased, the activation and the secretion of this cytokine are mediated by caspase-8, which was upregulated in PPMS patients alone.

Finally, IL-1β concentration was augmented in HC (median = 588 pg/ml) alone compared to MS patients (*p* < 0.05 vs. PPMS and BMS) (Figure 8D). These results confirm that, whereas IL-18 is elevated in MS, increased IL-1ß production is not always seen in this condition.

### Correlation Between Caspase-8 and Uric Acid Concentration

**Figure 9:**
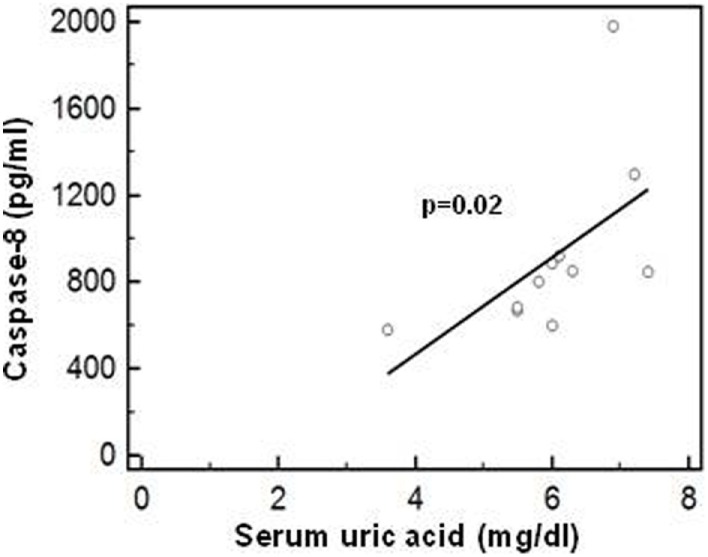
Correlation between caspase-8 and uric acid concentration. Correlation between active caspase-8 protein (lipopolysaccharide-primed and monosodium urate crystals-stimulated PBMCs) and serum uric acid concentration in PPMS patients. Statistical significance is shown.

Possible correlations between serum uric acid concentration and the production of the inflammasome proteins (caspase-1, caspase-8, IL-1β, IL-18, ASC-speck, and NLRP3) were analyzed next (data not shown). Results showed that in PPMS, i.e., in those patients in whom hyperuricemia was detected, a significant positive correlation between serum uric acid concentration and active caspase-8 protein is present (R*sp* = 0.811, *p* < 0.01) (Figure 9).

## Discussion

Multiple sclerosis is a neurodegenerative disease characterized by chronic inflammation; inflammasome-driven inflammation is postulated to play a role in MS, but as of today, there is no direct evidence that the NLRP3 inflammasome is involved in MS pathogenesis. MS is also characterized by a series of biochemical abnormalities that include increased serum and CSF concentrations of uric acid, a DAMP that is released from dying cells. Because the presence of hyperuricemia in MS is not confirmed by all authors, we first measured uric acid concentrations in serum of 41 MS patients with different disease phenotypes comparing the results to those of age- and sex-matched HCs. Results showed that hyperuricemia is seen in primary progressive MS (PPMS) confirming previous data demonstrating an increase of uric acid in the chronic phase of the disease (34).

Uric acid can act as a DAMP and stimulate the assembly of the inflammasome and, thus, can be one of the culprits for the activation of inflammasome-dependent inflammation in MS (9, 35). Hence, we next analyzed whether stimulation of PBMCs of MS patients with monosodium urate crystals (MSU) would result in the transcription of inflammasome-related proteins and the production of the pro-inflammatory cytokines that are the end products of the functional assembly of such proteins. Results showed that, whereas the expression of inflammasome proteins was upregulated upon MSU stimulation in cells of patients with all forms of active disease, the functional triad of inflammasome receptor, effector, and catalytic proteins as well as IL-18, were significantly upregulated in PPMS alone.

The NLRP3 inflammasome is suggested to play a pivotal role in development of MS-associated neuroinflammation. Thus, in the EAE animal model of MS, Nlrp3^−/−^ mice are either resistant to the development of EAE, or, develop a delayed disease that is characterized by reduced severity (14, 17). Notably, diminished Th1 and Th17 encephalitogenic responses, diminished inflammatory infiltrate, and reduced spinal cord demyelination and gliosis are seen as well in Nlrp3^−/−^ mice (14). In MS, results suggest the possible involvement of NLRP3 inflammasome in MS, but definite data are missing. Thus: (1) caspase-1, IL-1β, and IL-18 upregulation was shown to associate with the progression and severity of disease (17–19); and (2) caspase-1 expression was observed to be elevated in MS plaques (20) and in peripheral mononuclear cells of MS patients (19). Results herein indicate that fully functional inflammasome complexes are assembled in PPMS and are likely to drive inflammation in these patients.

Nod-like receptor protein 3 inflammasome assembly leads to the production of pro-inflammatory cytokines; IL-18, in particular, is an inflammasome-derived cytokine whose concentration was observed to be augmented in serum and CSF of MS patients (17, 22, 36). The possible involvement of this cytokine in the pathogenesis of MS was reinforced by the observations that IL-18 production by PBMCs of MS patients is increased (14) and that IL-18 is expressed by oligodendrocytes in brain tissues from patients with active MS (37). IL-18 plays an important role in Th1 response *via* its ability to induce IFN-gamma production in T lymphocytes and NK cells (38), and numerous reports confirm that Th1 responses directed toward self antigens are activated in MS (39–43). IL-18 is first synthesized as an inactive precursor; the precursor can be cleaved into the biologically active form of the protein, or can be stored intracellularly. Following its cleavage by caspases, the mature and biologically active form of IL-18 is secreted from monocytes/macrophages, even if over 80% of the IL-18 precursor remains unprocessed inside the cell. Secreted IL-18 initiates multiple signaling pathways and drives inflammatory responses, which result in neuronal injury or death (44–47). The canonical NLRP3 inflammasome requires caspase-1 activation for IL-1β and IL-18 processing. Recent results, nevertheless, indicate that T cell intrinsic inflammasome activity can drive IL-1β and IL-18 production *via* caspase-8 activation independently from caspase-1 activation (48, 49).

Our results showing that the expression of caspase-8 but not of caspase-1 is significantly upregulated in PPMS alone, strongly suggest that a caspase-1-independent ASC-NLRP3-caspase-8 inflammasome complex drives inflammation in PPMS patients.

Notably, IL-18 mRNA was significantly increased in MSU-stimulated cells of PPMS, AMS, and SMS patients compared to the values seen in BMS or in HC; IL-18 production, nevertheless, was not augmented in these patients. The most likely explanation for this apparent discrepancy is that caspase-1 was not modified in any of the groups examined and caspase-8, the “alternate” cleavage protein was augmented in PPMS alone; hence, only in these patients, the IL-18 precursor protein could be cleaved and the mature form of IL-18 could be secreted. Notably, assembly of functional NLRP3 inflammasomes did not result in significant changes in IL-1β mRNA expression and cytokine production in any of the groups examined. The existing literature on IL-1β expression in EAE and MS contains contradicting observations. Indeed: (1) most but not all authors have found increases of IL-1β in the CSF of MS patients (23–25) and (2) IL-1β staining was shown to be localized to resident microglia or differentiated macrophages but not to infiltrating monocytes, suggesting that IL-1β expression is induced within the CNS (50). Data herein seem to confirm these previous observations.

Taken together, our results suggest that in PPMS patients a possibly prolonged and chronic stimulation would result in the upregulation of mRNA expression of NLRP3, ASC, caspase-8, and IL-18 genes and of NLRP3, caspase-8, ASC-speck, and IL-18 pro-inflammatory cytokine; this could be justified by the observation that hyperuricemia is present in PPMS patients. Based on these data is tempting to speculate that, in PPMS, hyperuricemia would be responsible for the chronic activation of NLRP3 inflammasome and, as a consequence, the persistent activation of NF-Kb (51) and an excessive generation of pro-inflammatory cytokines. If this scenario is correct, therapeutic strategies aimed at reducing uric acid concentration and, as a consequence, downregulating inflammasome activity, might result in clinical benefit in these patients.

## Ethics Statement

The authors declare that the research was conducted in the absence of any commercial or financial relationships that could be construed as a potential conflict of interest.

## Author Contributions

FP, MS, and MC conceived and designed the research; FP, IM, FR, and MS performed the experiments; DC, LM, and MR are responsible for the clinical cohorts of patients; FP and MC analyzed the data and prepared the manuscript. All authors reviewed and approved the final manuscript.

## Conflict of Interest Statement

The authors declare that the research was conducted in the absence of any commercial or financial relationships that could be construed as a potential conflict of interest.

## Abbreviations

MS, multiple sclerosis; NLRP3, nod-like receptor protein 3; CSF, cerebrospinal fluid; MSU, monosodium urate crystals; RR, relapsing–remitting; A, acute; S, stable; PP, primary progressive; B, benign; HC, healthy control; IL, interleukin; CNS, central nervous system; DAMP, damage-associated molecular pattern; ASC, apoptosis-associated speck-like protein containing CARD; EAE, experimental autoimmune encephalomyelitis; MRI, magnetic resonance imaging; EDSS, expanded disability status scale.
